# Supplementary material for: Hi-reComb: constructing recombination maps from bulk gamete Hi-C sequencing
Source: Genetics. 2025 Jul 31;232(1):iyaf150. doi: 10.1093/genetics/iyaf150 (PMC7618151; doi:10.1093/genetics/iyaf150)
Supplement: iyaf150_Supplementary_Data [file iyaf150_supplementary_data.zip › Supplement Reference.docx]

Ronco F, Matschiner M, Böhne A, Boila A, Büscher HH, Taher AE, Indermaur A, Malinsky M, Ricci V, Kahmen A, et al. 2021. Drivers and dynamics of a massive adaptive radiation in cichlid fishes. Nature. 589(7840):76–81. doi:10.1038/s41586-020-2930-4.
