# Supplementary material for: Hi-reComb: constructing recombination maps from bulk gamete Hi-C sequencing
Source: Genetics. 2025 Jul 31;232(1):iyaf150. doi: 10.1093/genetics/iyaf150 (PMC7618151; doi:10.1093/genetics/iyaf150)
Supplement: iyaf150_Supplementary_Data [file iyaf150_supplementary_data.zip › Supplementary_Notes_and_Figures_GENETICS-2025-308324.pdf]

Supplementary Information for:

Hi-reComb: constructing recombination maps from bulk gamete Hi-C sequencing

Authors:
Milan Malinsky[1,2], Marion Talbi[1,2], Chenxi Zhou[3], Nicholas Maurer[4,5], Samuel Sacco[4,5], Beth Shapiro[4,5], Catherine L. Peichel[1], Ole Seehausen[1,2], Walter Salzburger[6], Jesse N. Weber[7], Daniel I. Bolnick[8], Richard E. Green[4,5] and Richard Durbin[8]

Affiliations:
[1] Institute of Ecology and Evolution, University of Bern, 3012 Bern, Switzerland
[2] Department of Fish Ecology and Evolution, EAWAG, 6047 Kastanienbaum, Switzerland
[3] Department of Genetics, University of Cambridge, Downing Street, Cambridge, CB2 3EH, UK
[4] Department of Biomolecular Engineering, University of California Santa Cruz, Santa Cruz, CA 95064, USA
[5] UCSC Genomics Institute, University of California Santa Cruz, Santa Cruz, CA 95064, USA
[6] Department of Environmental Sciences, Zoological Institute, University of Basel, 4051 Basel, Switzerland
[7] Department of Integrative Biology, University of Wisconsin-Madison, Madison, WI 53706, USA
[8] Department of Ecology and Evolutionary Biology, University of Connecticut, Storrs, CT 06269, USA

Correspondence to:
Milan Malinsky: millanek@gmail.com

Table of Contents

Supplementary Notes 2
Supplementary Note 1: Crossover likelihood calculation 2
Supplementary Note 2: Map inference and the EM procedure 4
Supplementary Figures 6
Supplementary Figure 1 6
Supplementary Figure 2 7
Supplementary Figure 3 8
Supplementary Figure 4 9
Supplementary Figure 5 10
Supplementary Figure 6 11
Supplementary Figure 7 12
Supplementary Figure 8 13

Supplementary Notes

Supplementary Note 1: Crossover likelihood calculation

Consider a pair of reads indicating by base calls at least two phased heterozygous sites that a crossover took place between them (i.e., $X = C$). Then let Q_{B1} be the (Illumina sequencer) base quality at the first heterozygous site, Q_{B2} the base quality at the second heterozygous site, Q_{P1} the haplotype phase quality at the first heterozygous site, and Q_{P2} the haplotype phase quality at the second heterozygous site. The Q_B and Q_P scores are Phred-scaled error probabilities. Therefore, we first obtain error probabilities: $E_{B1} = 10^{\frac{-Q_{B1}}{10}}$; $E_{B2} = 10^{\frac{-Q_{B2}}{10}}$; $E_{P1} = 10^{\frac{-Q_{P1}}{10}}$; $E_{P2} = 10^{\frac{-Q_{P2}}{10}}$. These quantities are used to calculate the crossover likelihood $l_{ci} = P(X = C \mid c_i = 1)$ for the read pair.

We consider four possibilities of how we could observe a read pair with $X = C$, given that a crossover really took place. We denote these components as l_{ci}^1, l_{ci}^2, l_{ci}^3, and l_{ci}^4. First, l_{ci}^1 corresponds to the case where both base calls and the haplotype phase at both sites are correct:

$$l_{ci}^1 = (1 - E_{B1}) * (1 - E_{B2}) * (1 - E_{P1}) * (1 - E_{P2})$$

Second, there is also a small possibility that a crossover took place if both base calls are incorrect, but haplotype phase calls are correct:

$$l_{ci}^2 = {E_{B1}}/{3} * {E_{B2}}/{3} * (1 - E_{P1}) * (1 - E_{P2})$$

We divide the base error probabilities E_{B1} and E_{B2} by three because the base calling errors must match the alleles present at the SNP and we assume that all three possible erroneous base calls are equally likely.

Third a crossover could take place if one base call is incorrect, combined with a phasing error at the same SNP:

$$l_{ci}^3 = {E_{B1}}/{3} * (1 - E_{B2}) * E_{P1} * (1 - E_{P2}) + (1 - E_{B1}) * {E_{B2}}/{3} * (1 - E_{P1}) * E_{P2}$$

Fourth, it is possible that a crossover took place if there are phasing errors on both SNPs:

$$l_{ci}^4 = (1 - E_{B1}) * (1 - E_{B2}) * E_{P1} * E_{P2}$$

Finally, we obtain the overall crossover likelihood as the sum of these probabilities:

$$l_{ci} = l_{ci}^{1} + l_{ci}^{2} + l_{ci}^{3} + l_{ci}^{4}$$

Supplementary Note 2: Map inference and the EM procedure

For each chromosome, `Hi-reComb` produces a recombination map that integrates crossover probabilities from all informative read pairs mapping onto that chromosome. The procedure is similar to the algorithm used by Halldorsson et al. (2019)(Halldorsson et al. 2019). Let $\mathbb{P}(c_i)$ be the crossover probability for a read pair i between a pair of defining phase-informative SNPs at physical positions along the chromosome g_{li} and g_{ui}. We use d_i to denote the physical distance between g_{li} and g_{ui}. The per-bp crossover probability p_i^{bp} for the read pair i is then $p_i^{bp} = \mathbb{P}(c_i)/d_i$. Next, let $(g_j)_0^N$ be the ordered sequence of defining SNPs for all informative read pairs and, within this sequence, let l_i and u_i be the indices of SNPs that define the informative read pair i.

We estimate the per-bp recombination rate r_j^{bp} for each genomic interval $I_j = [g_j, g_{j+1})$. We initialize the genetic map by finding all the K read-pairs that span each I_j and setting $r_j^{bp} = \sum_{k=1}^{K} p_k^{bp} / \sum_{k=1}^{K}(1 - \mathbb{P}(c_k))$. The proportion of recombination expected to occur within the interval, the recombination fraction r_j, is estimated as $r_j = r_j^{bp} * (g_{j+1} - g_j + 1)$. The sequence of recombination rate values $(r_j^{bp})_0^{N-1}$ specifies the entire genetic map m on the interval $[g_0, g_N)$.

We use the initial genetic map as an input into the EM procedure. First, we calculate for each read pair with positive crossover probability (i.e., $p_i^{bp} > 0$), the probability that the crossover occurred within a genomic interval I_j. This probablity p_i^j depends on the recombination rates in all the intervals that are covered by the read pair; specifically:

$$p_i^j = \frac{r_j}{\sum_{k=l_i}^{u_i} r_k}$$

An updated genetic map is calculated from these probabilities, with recombination fraction values given by $r_j = \sum_{k=1}^{K} p_k^j / \sum_{k=1}^{K}(1 - \mathbb{P}(c_k))$, where the sums are again over all the K read-pairs that span the interval I_j. With the updated map, we then calculate new values for p_i^j then update genetic map again, and continue for `--maxEM` iterations or until convergence is reached, as

defined by $\sum_{j=0}^{N-1} \left| r_j^m - r_j^{m-1} \right| < \varepsilon$, where the superscript m denotes iteration number m and ε is provided by the user via the `--epsilon` parameter.

Supplementary Figures

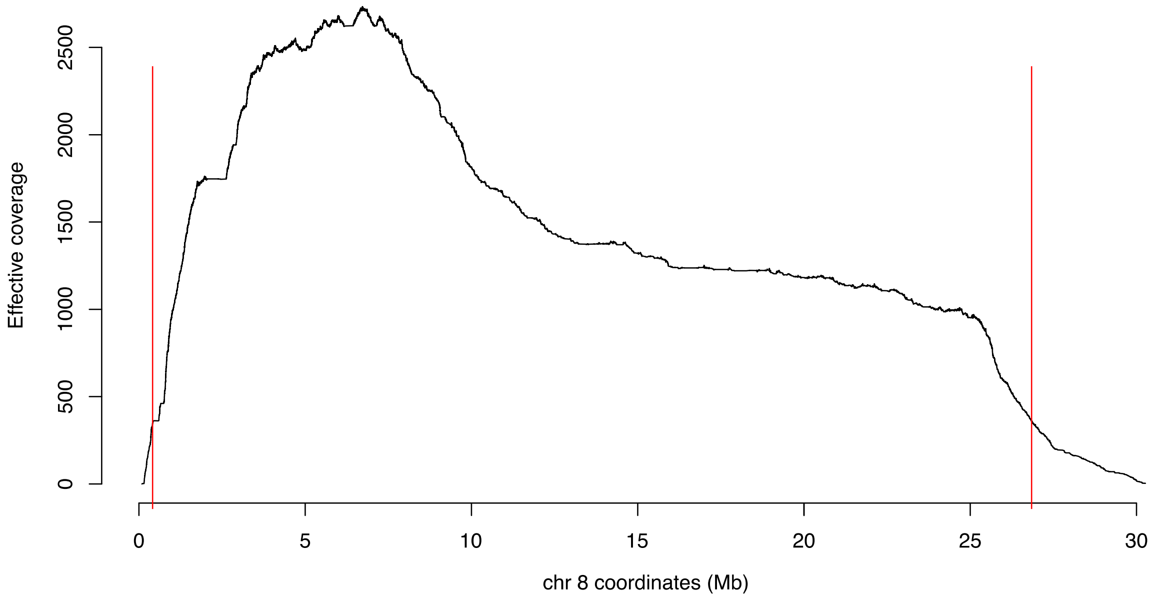

Supplementary Figure 1: Effective coverage along the chromosome and the 'edge effect'.

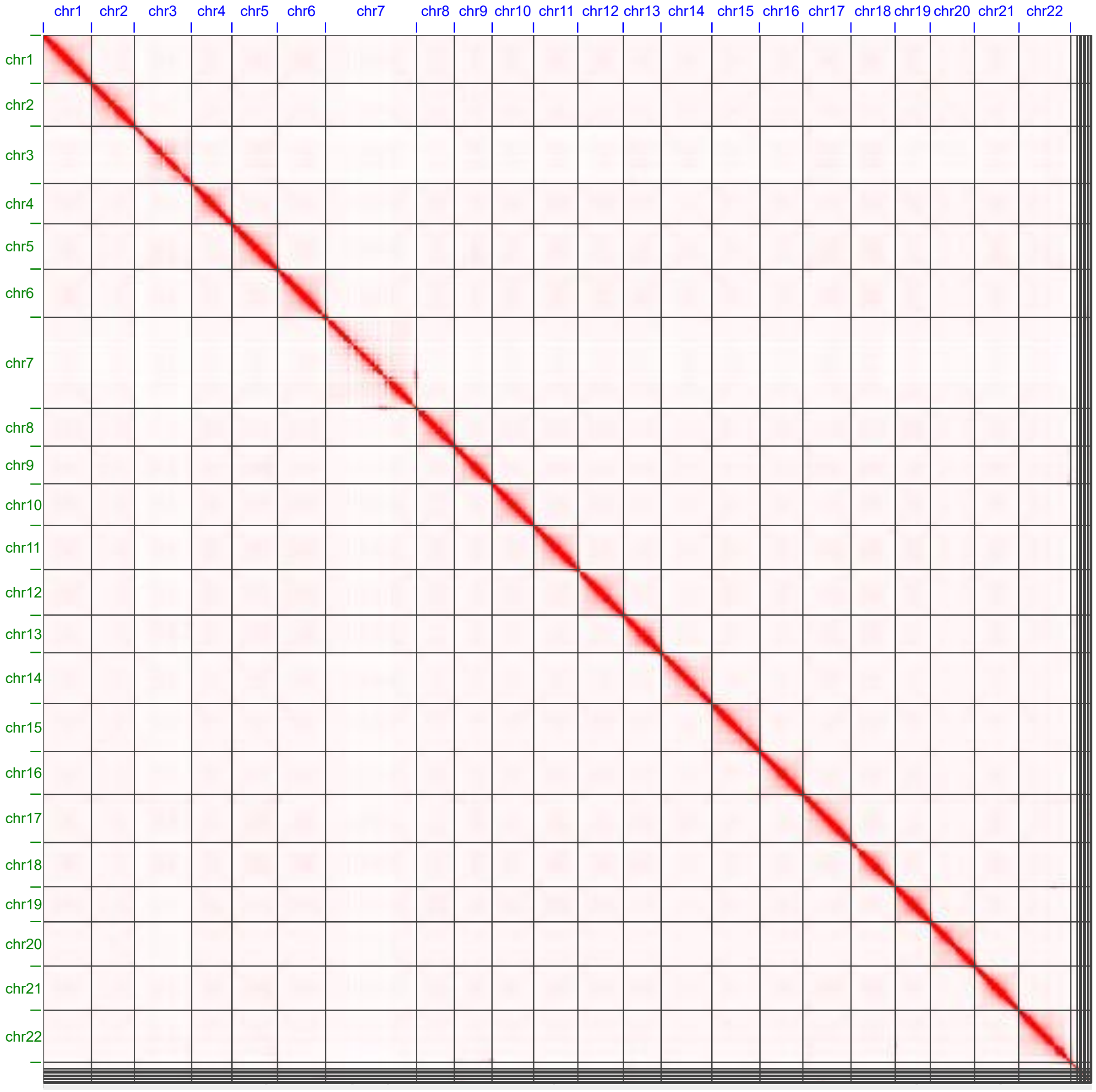

Supplementary Figure 2: Hi-C contact map for read pairs mapped to the newly scaffolded *Neolamprologus multifasciatus* fNeoMul1.2 reference genome.

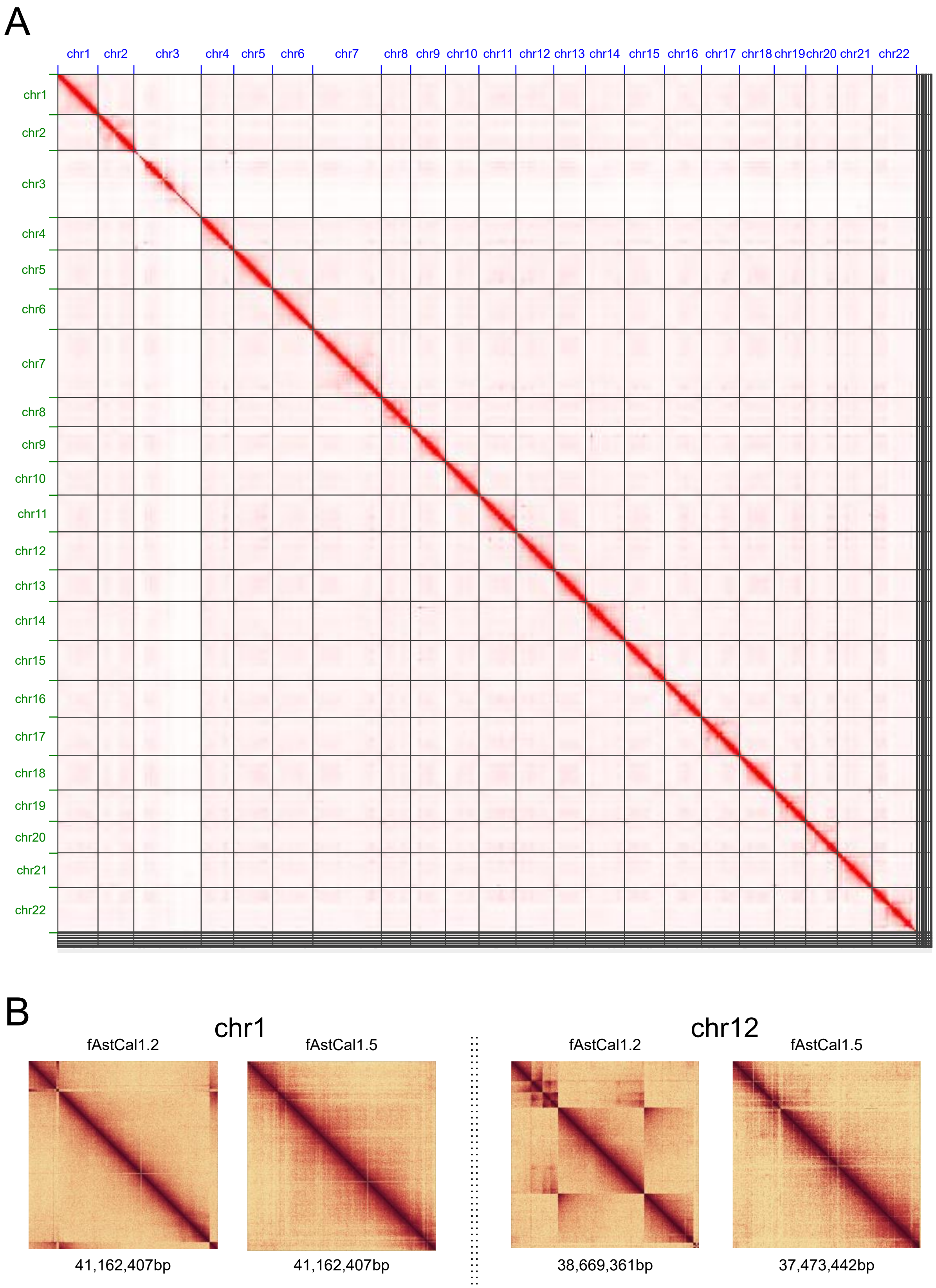

Supplementary Figure 3: (A) Hi-C contact map for read pairs mapped to the newly scaffolded *Astatotilapia calliptera* fAstCal1.5 reference genome. **(B)** A comparison of the original fAstCal1.2 vs. the new fAstCal1.5 assemblies for two example chromosomes, showing the resolution of disagreements between the Hi-C contact map and the original assembly.

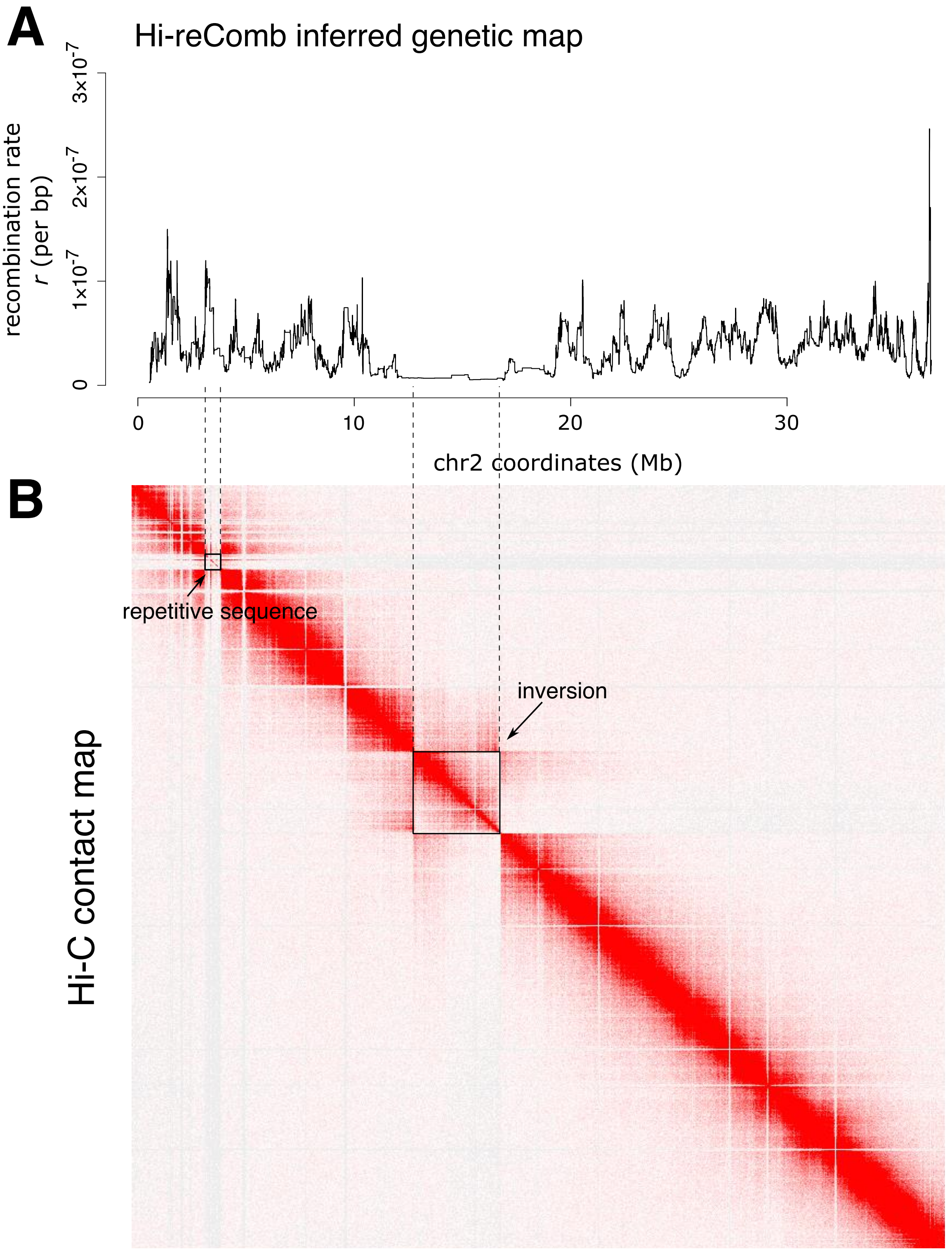

Supplementary Figure 4: An inversion and suppressed recombination on *A. stuartgranti* chromosome 2. (**A**) The `Hi-reComb` inferred genetic map. (**B**) Hi-C contact map. Both panels are aligned along chr2 coordinates of the *A. calliptera* fAstCal1.5 reference. The figure illustrates a useful advantage of the Hi-C approach: at the same time as being used for recombination inference, the Hi-C contact map provides information about structural variation in the donor individual. Thus, we can see that the region of low recombination in the centre of the chromosome corresponds to and extends beyond the inversion, although the inversion relative to the reference assembly appears to be homozygous and the mechanism of recombination suppression is not clear.

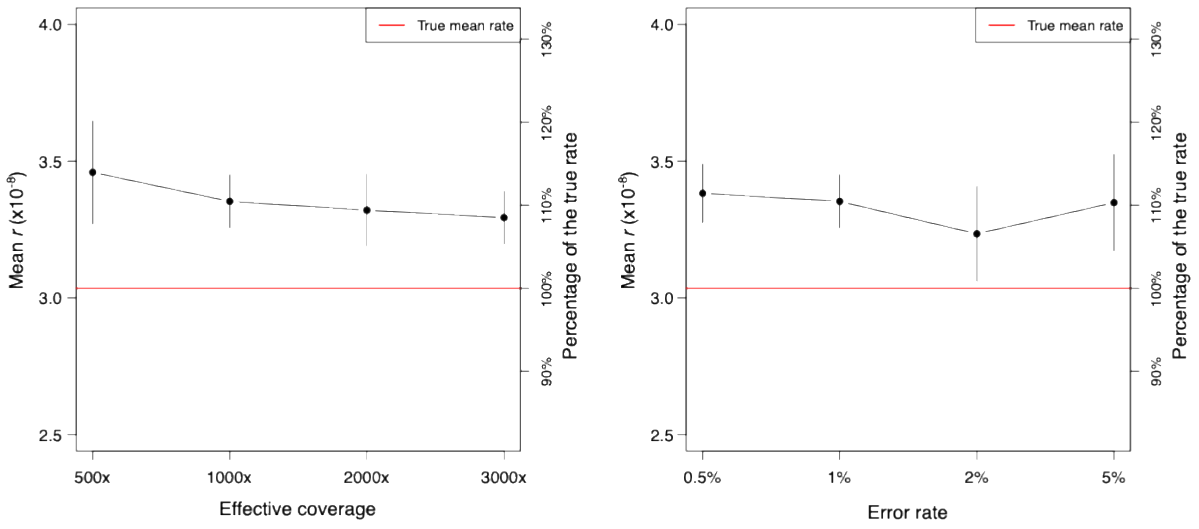

Supplementary Figure 5: The extent of overestimation of mean *r* in maps reconstructed from simulations. Higher effective coverage leads to more accurate estimates, while error rate does not seem to have any impact.

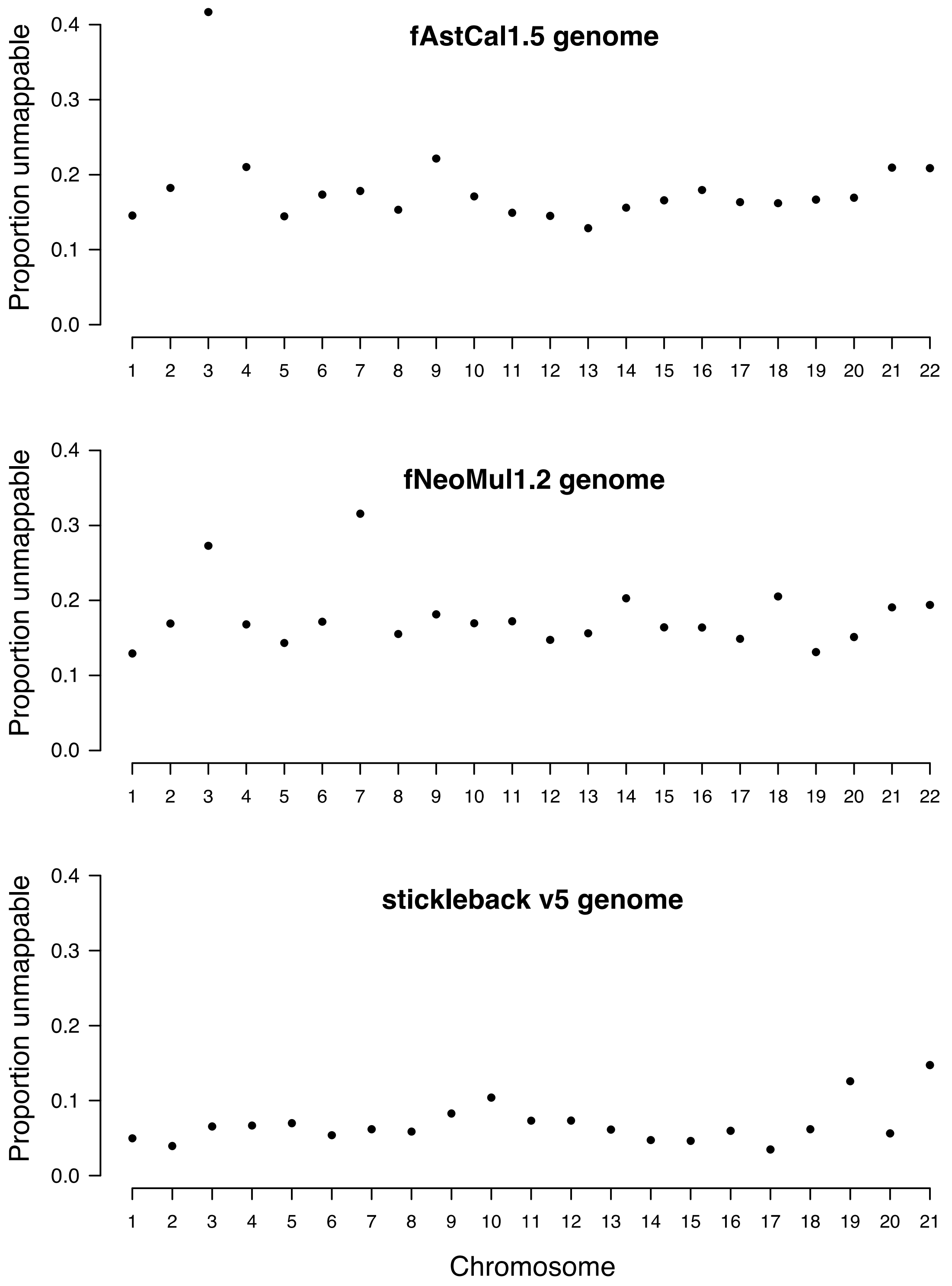

Supplementary Figure 6: The proportion of unmappable sequence on different chromosomes in the three genome asseblies used in this study. Unmappable sequences are defined as regions covered by the mappability mask (see **Methods**). In the fAstCal1.5, chromosome 3 has by far the most unmmapable sequence (42%), while the next highest number is 22% on chromosome 9. It is also notable that the stickleback assembly contains much less unmappable sequence than the cichlid genomes (stickleback mean: 6.9%; cichlid 17.7% in fNeoMul1.2 and 18.2% in fAstCal1.5).

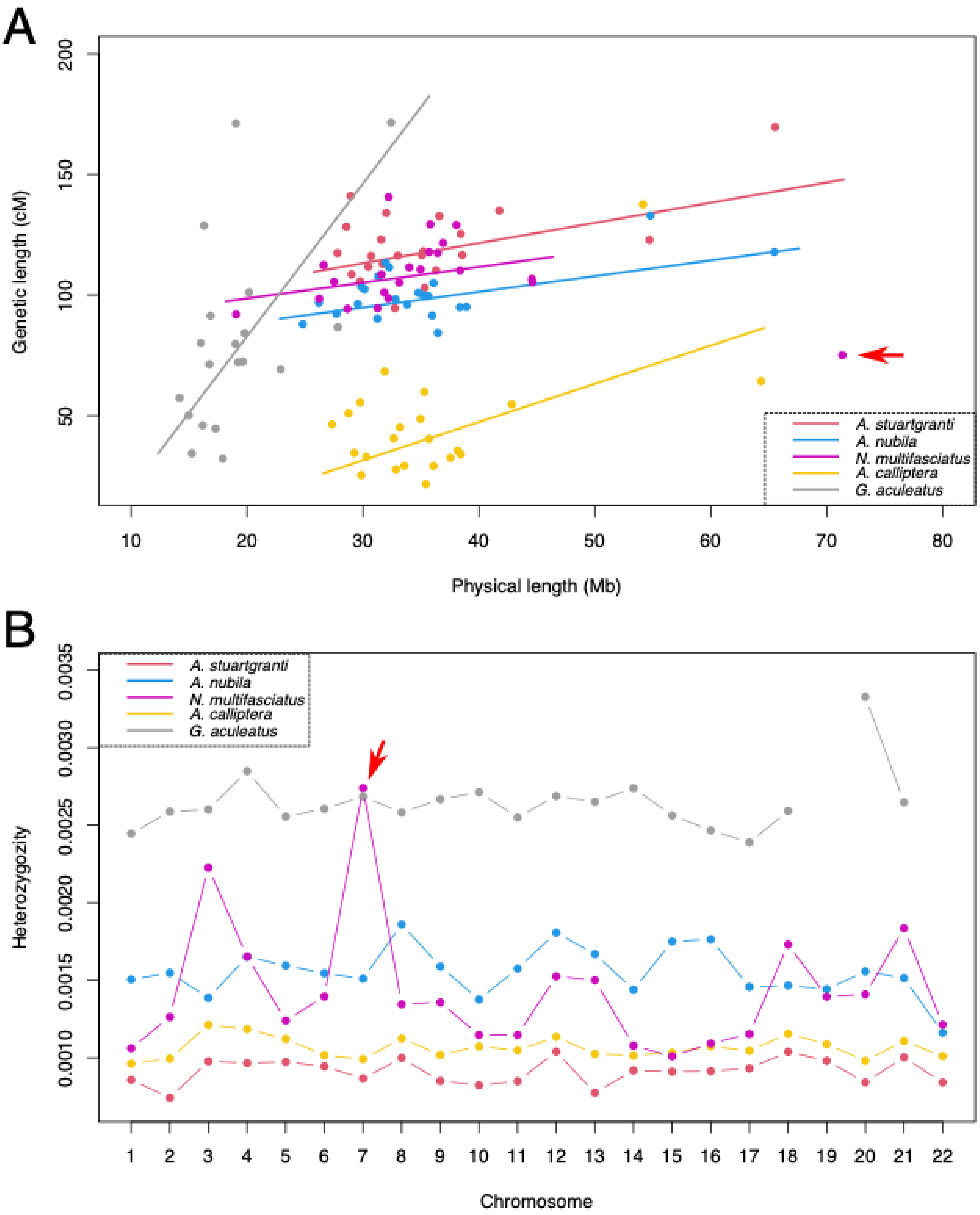

Supplementary Figure 7: Per chromosome statistics. (A) The positive relationship between physical and genetic map length in each species. A *N. multifasciatus* outlier chromosome (chr 7) is highlighted by a red arrow. Note that several A. calliptera and and G. aculeatus chromosomes have estimated map lengths below 50 cM (see main text for details). **(B)** Heterozygozity (phased SNPs only) across chromosomes. The level of heterozygozity in *N. multifasciatus* varies much more than in other species, with chr 7 being a clear outlier. This variation could be explained by segregating admixed ancestry, consistent with previous reports *(Ronco et al. 2021)*. The presence of heterozygous haplotypes of different ancestries and incompatibilities between them in *N. multifasciatus* could be responsible for the relatively low recombination rate on chr 7.

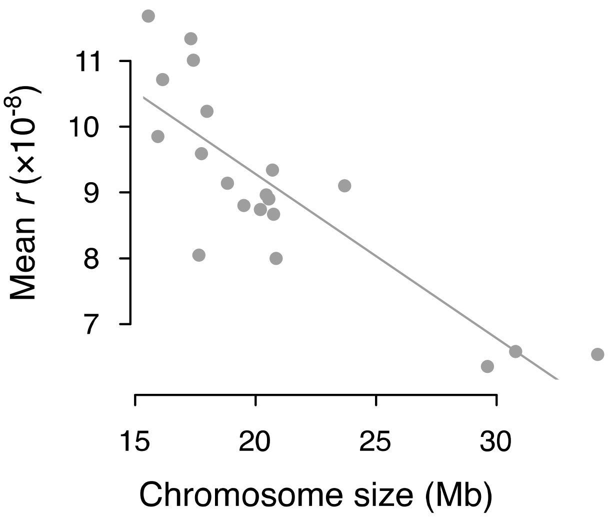

Supplementary Figure 8: The negative relationship between the chromosome size and the mean recombination rate in stickleback. These results reflect recombination maps based on trio-phased data.
